# The functional sites of miRNAs and lncRNAs in gastric carcinogenesis

**DOI:** 10.1007/s13277-015-3136-5

**Published:** 2015-02-01

**Authors:** Xiangxiang Wan, Xiaoyun Ding, Shengcan Chen, Haojun Song, Haizhong Jiang, Ying Fang, Peifei Li, Junming Guo

**Affiliations:** 1 Department of Gastroenterology, Ningbo First Hospital, No. 59 Liuting Street, Ningbo, 315010 China; 2 Department of Biochemistry and Molecular Biology, Zhejiang Provincial Key Laboratory of Pathophysiology, Ningbo University School of Medicine, Ningbo, 315211 China

**Keywords:** miRNAs, lncRNAs, Gastric carcinogenesis, Mechanisms

## Abstract

Gastric cancer is one of the most common malignant diseases and has one of the highest mortality rates worldwide. Its molecular mechanisms are poorly understood. Recently, the functions of non-coding RNAs (ncRNAs) in gastric cancer have attracted wide attention. Although the expression levels of various ncRNAs are different, they may work together in a network and contribute to gastric carcinogenesis by altering the expression of oncogenes or tumor suppressor genes. They affect the cell cycle, apoptosis, motility, invasion, and metastasis. Dysregulated microRNAs (miRNAs) and long non-coding RNAs (lncRNAs), including miR-21, miR-106, H19, and ANRIL, directly or indirectly regulate carcinogenic factors or signaling pathways such as PTEN, CDK, caspase, E-cadherin, Akt, and P53. Greater recognition of the roles of miRNAs and lncRNAs in gastric carcinogenesis can provide new insight into the mechanisms of tumor development and identify targets for anticancer drug development.

## Introduction

Gastric cancer (GC) is the fourth most frequent malignancy, with most patients being diagnosed in advanced stages with limited treatment options. GC continues to present a major clinical challenge [1] and ranks as the second leading cause of cancer-related death [2]. The development of GC is a complex and multistep process. It results from a combination of environmental factors and the accumulation of generalized and specific genetic alterations. Predisposing factors include *Helicobacter pylori* (*H. pylori*) infection, high salt intake, smoking, and familial genetic components [1].

Non-coding RNAs (ncRNAs) are generally divided into three major classes based on the following sizes : (1) short ncRNAs, including the much-studied microRNAs (miRNAs), which mediate posttranscriptional gene silencing, and Piwi-interacting RNAs (piRNAs); (2) mid-size ncRNAs, such as small nucleolar RNAs (snoRNAs); and (3) long non-coding RNAs (lncRNAs), which act as signals, guides, or scaffolds to chromatin to regulate the expression of target genes [3].

Over the past few years, increasing studies have demonstrated that miRNAs [4] and lncRNAs [5] could function as oncogenes or tumor suppressor genes. In GC, many miRNAs and lncRNAs are dysregulated and can regulate gene expression and biological functions cooperatively. Some play key roles in cellular processes including the cell cycle, apoptosis, and metastasis [2, 6].

## ncRNAs associated with the cell cycle in GC

**Fig 1:**
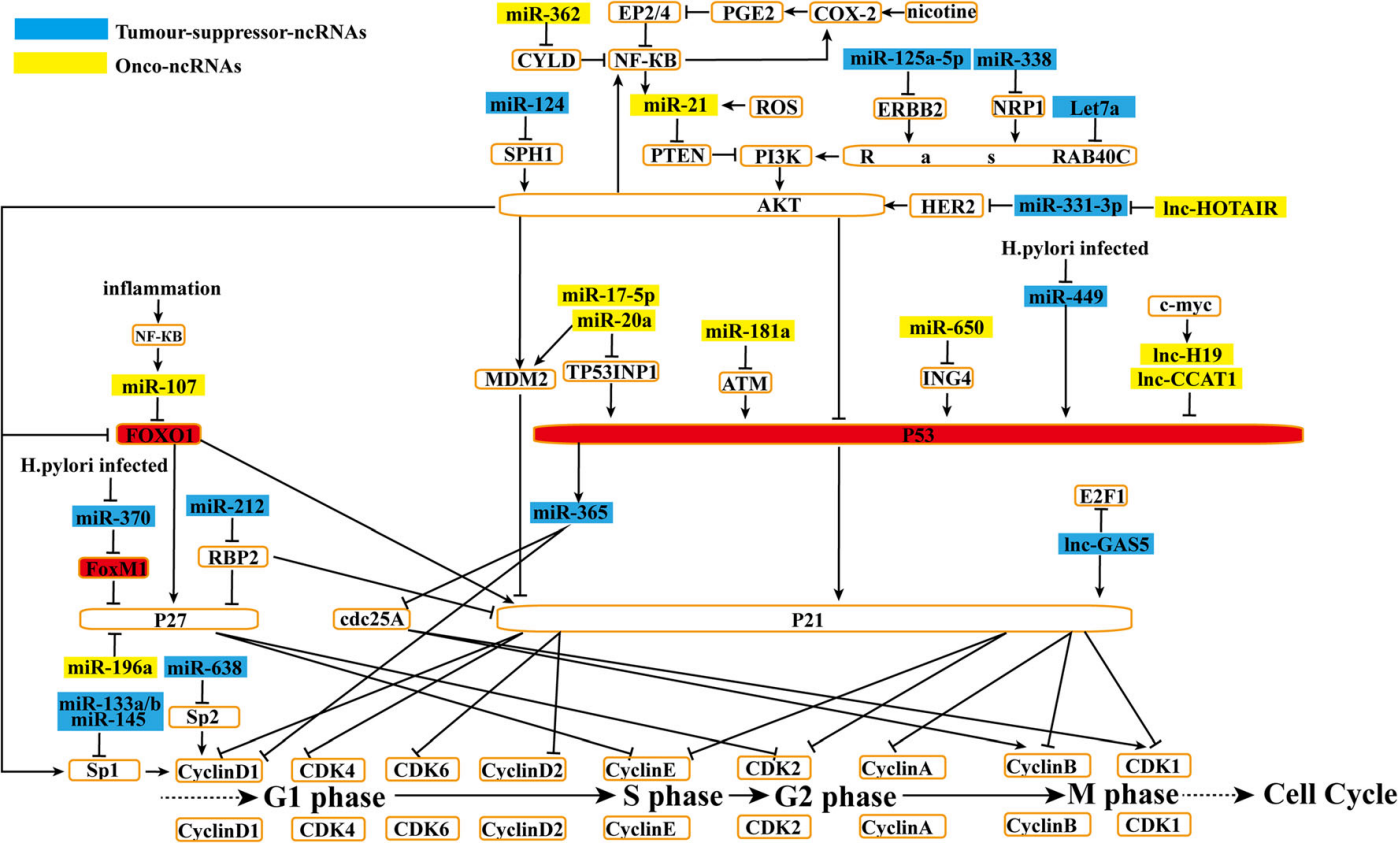
ncRNAs affect Akt pathway in cell cycle. MiRNAs and lncRNAs affect gastric cancer cell cycle progression. By regulating Akt pathway, onco-ncRNAs promote gastric cancer cell growth. Conversely, tumor suppressor ncRNAs suppress the gastric cancer cell cycle

**Fig 2:**
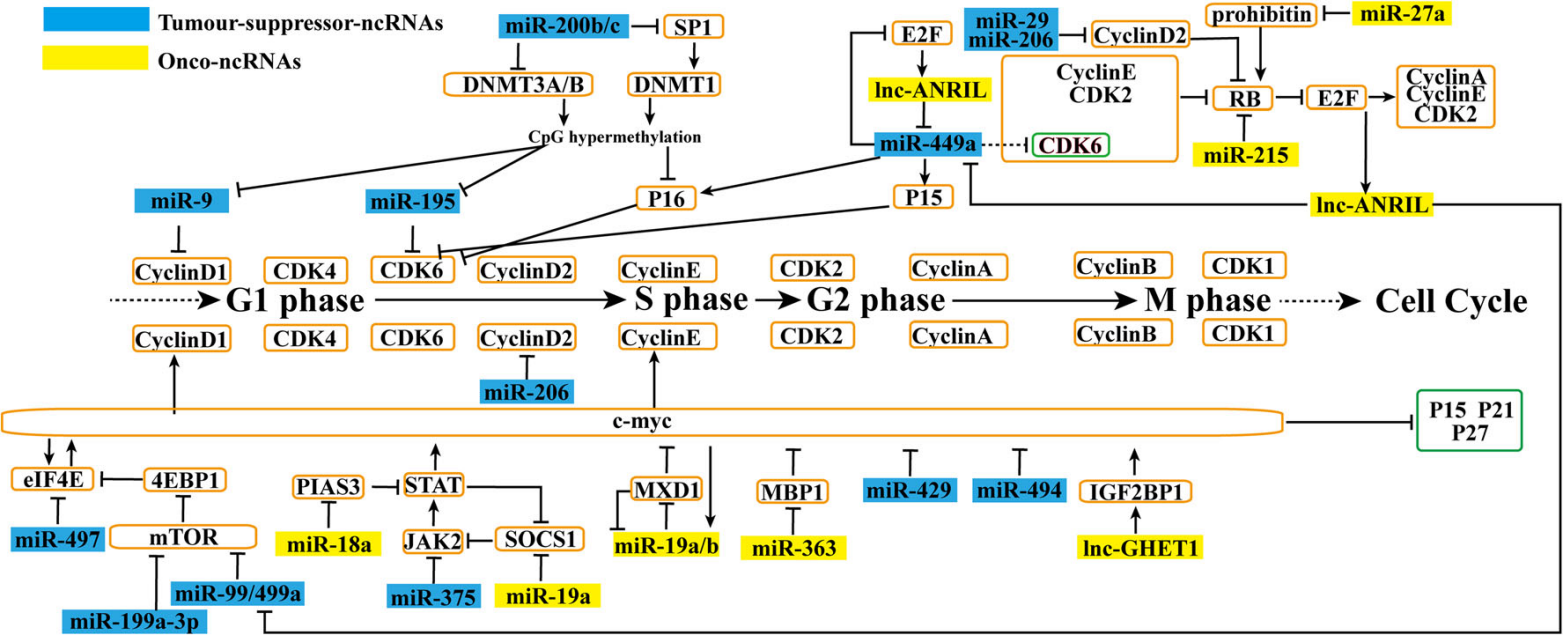
ncRNAs affect CDKIs and Myc pathway in cell cycle. MiRNAs and lncRNAs affect gastric cancer cell cycle progression. By regulating CDKIs and their downstream and Myc pathway, onco-ncRNAs promote gastric cancer cell growth. Conversely, tumor suppressor ncRNAs suppress the gastric cancer cell cycle

The cell cycle includes four phases: (1) G1 (Gap1), (2) S (DNA synthesis), (3) G2 (Gap2), and (4) M (mitosis). Initiation of each phase requires Cyclin/Cyclin-dependent kinase (CDK) complexes, which are assisted by several protein kinases [7]. In late G1, Cyclin D-CDK4/6 activity begins to decrease, and Cyclin E-CDK2 activity rises. Cyclin E-CDK2 can increase E2F by inhibiting Rb (retinoblastoma), which hampers E2F, and upregulating a number of targets important for S-phase entry and progression. During early S-phase, with the decomposition of Cyclin E, Cyclin A complexes with CDK2 to drive progression through S-phase into G2. From mid-G2 onwards, CDK2 activity decreases and Cyclin A associates with CDK1. Finally, to enter M-phase, Cyclin B complexes with CDK1 and phosphorylates their targets. In late M-phase, following cytokinesis, Cyclin B is degraded, indicating the start of the next round of the cycle [8]. CDK activity is modulated by CDKs including CAK (a complex of CDK7 and Cyclin H) by CDK phosphatases (CDC25 phosphatases) that activate the Cyclin B/CDK1 complex to promote mitotic entry, and by CDK inhibitors (CDKIs) including the Ink4 (p15^Ink4b^, p16^Ink4a^, p18^Ink4c^, and p19^Ink4d^) and Cip/Kip (p21^Cip1^, p27^Kip1^, and p57^Kip2^) families [9]. As p53 upregulates p21^Cip1^, p53-mediated tumor suppressor pathways also block the cell cycle [8]. Myc can both activate and repress the expression of *Cyclin* and *CDK* genes [9]. Interestingly, in GC, miRNAs and lncRNAs are associated with almost all the cell cycle regulatory sites. (Figs. 1 and 2)

### ncRNAs affect the Akt pathway in the cell cycle

#### Effect upstream of the Akt axis

The Akt-FOXO1-p21^Cip1^/p27^Kip1^ axis plays an important role in the cell cycle (Fig. 1). Akt activation leads to the inhibition of FOXO1 and, consequently, downregulates the expression of p21^Cip1^ and p27^Kip1^. Decreased miR-124 in GC can inhibit the cell cycle by downregulating SPHK1, which triggers this axis [10]. Another important cell cycle signaling pathway is the PI3K-Akt-p53-Cyclin D1/cdc25A axis, which is induced by PTEN deficiency. As the intermediate link, miR-365 is indirectly suppressed by Akt by decreasing p53 abundance. In turn, reduced miR-365 leads to the upregulation of Cyclin D1 and cdc25A [11]. In addition to miR-124 and miR-365, many other miRNAs and lncRNAs may influence the cell cycle by regulating upstream components of the Akt pathway in GC (Fig. 1). miR-21 promotes cell proliferation by targeting PTEN [12]. PTEN downregulation promotes Akt signaling and results in increased NF-κB [13]. In GC, both ROS [14] and NF-κB [15] can increase the level of miR-21. Meanwhile, miR-362 upregulation activates NF-κB signaling by repressing CYLD [16]. In addition, nicotine enhances the binding of NF-κB to the miR-21 promoter. The activation of COX-2/prostaglandin E2 (PGE2) signaling in response to nicotine is mediated by the action of the prostaglandin E receptors (EP2 and EP4), which impair nicotine-mediated NF-κB activity [15]. Furthermore, activated NF-κB upregulates the expression of Cox-2 [13], thus forming a complex regulatory network among miR-21, Akt, and NF-κB in the progress of gastric carcinogenesis. Receptor tyrosine kinases (RTKs) regulate a key initiator of phosphoinositide-3 kinase (PI3K)-Akt through the RAS signaling pathway [17]. As an RTK, ERBB2 is upregulated and inversely correlated with miR-125a-5p expression in GC. Both ERBB2 and its primary downstream signaling pathway through Akt are suppressed by miR-125a-5p [18]. miR-338 reduced in GC decreases Akt phosphorylation by attenuating the expression of NRP1, a receptor for the vascular endothelial growth factor (VEGF) isoform VEGF-165 [19]. Let-7a decreased in GC regulates the cell cycle by directly downregulating RAB40C, a member of the RAS family [20]. HOTAIR [21] is upregulated in GC and inversely correlates with miR-331-3p. By binding miR-331-3p, HOTAIR acts as a competing endogenous RNA (ceRNA), thus abolishing the miRNA-induced repressive activity on HER2, which promotes GC cell growth through the Akt pathway [22].

#### Effect downstream of the Akt axis

As for the downstream of the **Akt** axis, other miRNAs and lncRNAs can affect FOXO1 or p53 directly or indirectly (Fig. 1). Li et al. suggested that in GC, the NF-κB-dependent upregulation of miR-107 could inhibit FOXO1 protein expression and induce proliferation [23]. However, Li et al. reported the converse result, that miR-107 might act as a tumor suppressor by directly targeting CDK6 to block the GC cell cycle [24]. Different from FOXO1, another member of the FOX family, FOXM1, decreases the activity of p27^Kip1^. It is negatively regulated by miR-370 and reduced by Hp infection in GC [25]. Like the FOX family, p53 is influenced by many ncRNAs. Tony et al. found that Hp indirectly modulated p53 and its downstream target p21 by downregulating miR-449 [26]. miR-650 upregulation in GC targets *ING4*, which is thought to enhance p53 function in gene transcription and promote cell growth [27]. miR-181a acts as an oncomir in GC by targeting the tumor suppressor gene *ATM* [28], which increases the expression and activity of p53 [29]. TP53INP1 is a key element in p53-mediated cell death and cell cycle arrest. Both miR-17-5p and miR-20a are upregulated in GC and can promote cell growth via deregulating TP53INP1 and P21. However, miR-17-5p/20a function independently on p53. They also inhibit p21 indirectly by increasing murine double minute 2 (MDM2), a negative regulator of p21 [30], which is also promoted by PI3K/Akt signaling in GC [31]. H19 [32] and CCAT1 [33] are c-Myc-induced ncRNAs. Both can decrease the activity of p53 [33, 34]. Furthermore, upregulated H19 promotes miR-675, which inhibits the tumor suppressor runt domain transcription factor1 (RUNX1) when promoting GC cell growth [35].

### ncRNAs affect CDKIs and their downstream in cell cycle

In addition to the classical Akt pathway, many ncRNAs can directly or indirectly regulate P27, P21, P16, P15 or their downstream targets (Figs. 1 and 2). Sun et al. demonstrated that upregulated miR-196a [36] could inhibit p27^Kip1^ expression, which prevents cell cycle progression by inhibiting Cyclin E/CDK2 activity. And the suppressive expression of GAS5 resulted in a decrease of P21 and an increase in E2F1 and Cyclin D1 [37] (Fig. 1). miR-212 inhibits proliferation and increases the expression of P21^Cip1^ and P27^Kip1^ indirectly by repressing retinoblastoma-binding protein 2 (RBP2) [38] (Fig. 1). Both miR-200b and miR-200c are downregulated in GC and can target DNMT3A/B directly or downregulate DNMT1 indirectly through mediating the decrease of specificity protein 1 (Sp1) (Fig. 2). Decreased DNMTs result in DNA hypomethylation, which is responsible for the overexpression of p16 [39]. Upregulated ANRIL suppresses the expression of miR-449a, p15^Ink4B^, and p16^Ink4A^. The downregulation of miR-449a releases CDK6. At the same time, the lower expression of p15^Ink4B^ and p16^Ink4A^ reduces their inhibitory effect on CDK6 (Fig. 2). Therefore, all this abnormal expression will promote CDK6. Increased CKD6 can inhibit Rb, thus releasing E2F1. In turn, the released E2F1 increases *ANRIL* expression, forming a positive feedback loop and continuously promoting GC cell proliferation [40]. Complementary to this, both miR-29 [41] and miR-206 [42] act as tumor suppressors by targeting *Cyclin D2*, which regulates the cell cycle by controlling Rb phosphorylation levels (Fig. 2). In addition, overexpressed miR-215 [43] and miR-27a [44] regulate Rb directly and indirectly, respectively, via targeting *prohibitin* genes (Fig. 2). In addition to Cyclin D2, Cyclin D1, which also promotes cell cycle progression by activating CDK4/6, is another important target. Both Sp1 [45] and Sp2 [46] can increase the expression of Cyclin D1. Qiu [45] and Zhao [46] et al. reported that miR-145, miR-133a, and miR-133b could decrease Sp1, and miR-638 could inhibit Sp2. In GC, Sp1 is also regulated by PI3K-Akt signaling [47] (Fig. 1). Furthermore, hypermethylation-mediated silencing of miR-9 [48] directly causes increased Cyclin D1 expression [49]. CDK is also regulated by miRNAs. miR-195 silencing by DNA hypermethylation negatively regulates the expression of CDK6 by binding the *CDK6* mRNA 3′-UTR [50] (Fig. 2).

### ncRNAs affect the Myc pathway in the cell cycle

Myc is another important functional site in cell cycle. On one hand, it directly induces Cyclin D or Cyclin E expression. On the other hand, it indirectly promotes the cell cycle by inhibiting p15^INK4^, p27^Kip1^, and p21^CIP1^ [51]. Interestingly, Myc is also regulated by many ncRNAs in GC (Fig. 2). c-Myc and eIF4E can promote each other [52]. Meanwhile, eIF4E is increased by mTOR, which phosphorylates 4E-BP1 [53], and inhibited by miR-497 [54]. mTOR is directly inhibited by miR-199a-3p [55] and abrogated by miR-99a/miR-499a, which are epigenetically inhibited by ANRIL [40]. c-Myc is a STAT3-mediated gene, which is negatively regulated by protein inhibitor of activated signal transducer and activator of transcription 3 (PIAS3). miR-18a indirectly modulates c-Myc by targeting PIAS3 [56]. Suppressors of cytokine signaling (SOCS) family proteins are important negative feedback inhibitors of JAK/STAT [57]. miR-375 [58] can repress JAK2, and miR-19a [57] can downregulate SOCS1. Therefore, all three miRNAs may ultimately affect the GC cell cycle through regulating c-Myc. c-Myc also acts as a transactivator of miR-19a/b, which inhibits MXD1 expression. In turn, downregulated MXD1 loses its inhibitory effect on miR-19a/b and c-Myc. The direct association between miR-19a/b and the c-Myc antagonist gene *MXD1* indicates a positive feedback loop between the three [59]. Upregulated miR-363 promotes GC cell growth by suppressing c-Myc promoter binding protein 1 (MBP-1), which initiates the specific inactivation of Myc [60]. In addition to these indirect effects, some miRNAs regulate c-Myc directly in GC. Both the downregulation of miR-429 [61] and miR-494 [62] cause increased c-Myc. In addition to miRNAs, GHET1 promotes the stability and expression of c-Myc by interacting with insulin-like growth factor 2 mRNA binding protein 1 (IGF2BP1) [63].

## ncRNAs associated with apoptosis in GC

**Fig 3:**
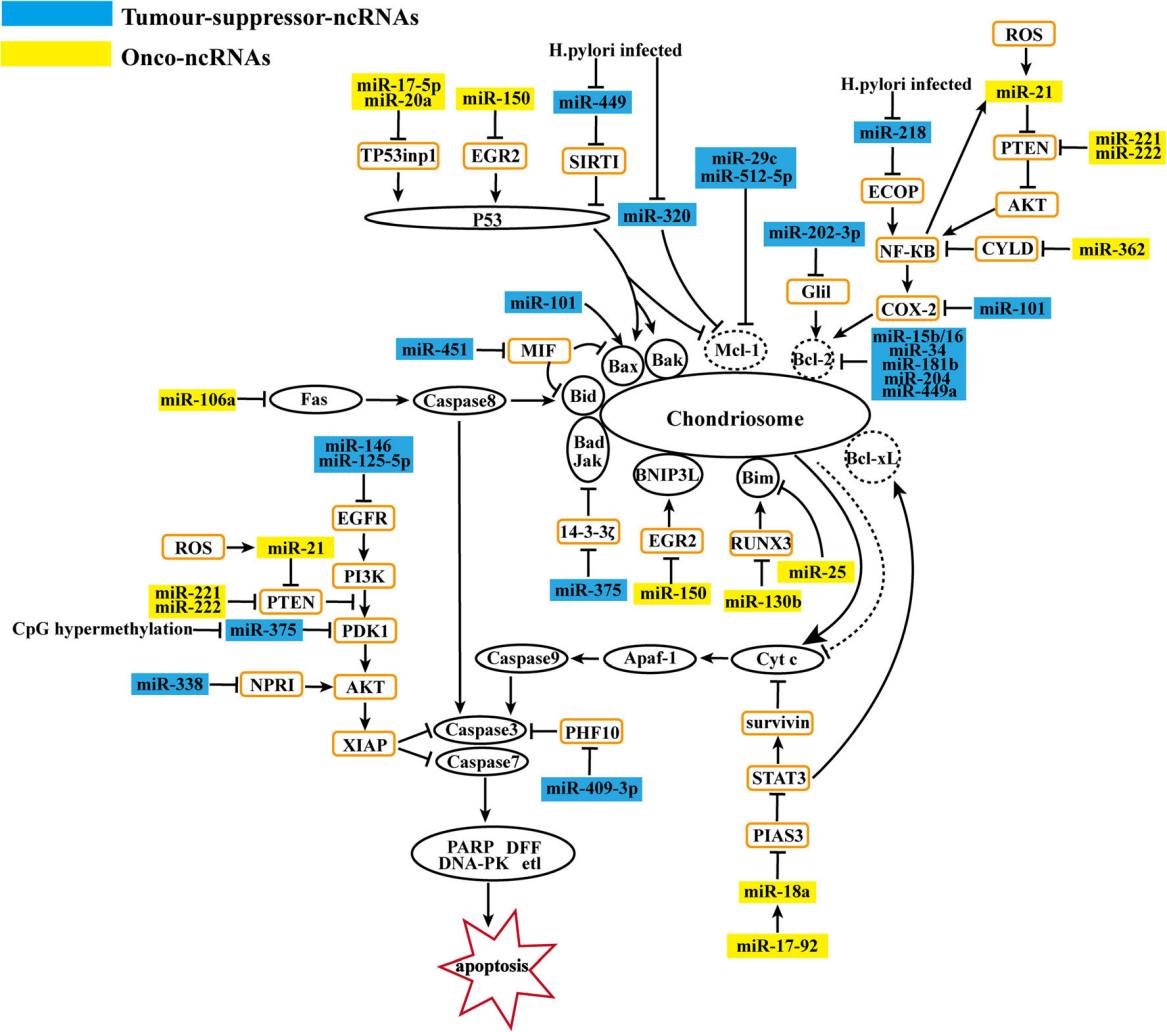
ncRNAs associated with apoptosis in GC. MiRNAs and lncRNAs affect gastric cancer cell apoptosis. By regulating extrinsic and intrinsic pathways, onco-ncRNAs suppress gastric cancer apoptosis. Conversely, tumor suppressor ncRNAs promote gastric cancer cell apoptosis

Apoptosis is an intrinsic cellular suicide program. Its initiation and progress are accurately regulated by upstream regulators and downstream effectors. Caspases are generally categorized as initiators (caspase-2, -8, -9, -10) and effectors (caspase-3, -6, -7). These initiators activate apoptosis through the following three signaling pathways: (1) the death receptor pathway (extrinsic pathway), (1) the mitochondrion pathway (intrinsic pathway), and (3) the endoplasmic reticulum pathway [64, 65]. The extrinsic apoptotic program contains the Fas ligand/Fas receptor and Apo3. For example, the Fas-associated death domain protein (FADD) directly binds to the Fas death domain and activates caspase-8, leading to cell death [65]. For the intrinsic pathway, the regulators and effectors are controlled by counterbalancing the Bcl-2 family, including the proapoptotic (Bax, Bak, Bok, Bim, Bid, Bad, Bmf, Bik, BNIP3L, Noxa, Puma, and Hark) and antiapoptotic (Bcl-2, Bcl-xL, Bcl-w, Mcl-1, and Al/Bfl-1) members [2]. These dysregulated apoptotic molecules can change the permeability of the mitochondrial membrane and the release of cytochrome C (Cyt c) and other proteins. Cyt c can raise the intracellular quality of caspase-9 precursors and promote self-activation, starting a caspase cascade and activating downstream caspase-3 and caspase-7, which cause apoptosis. All these pathways associate with each other and coordinately regulate apoptosis [65] (Fig. 3).

### ncRNAs affect apoptosis initiators

Altered expression of miRNAs can affect the progress of apoptosis in GC cells [2, 6]. (Fig. 3). First, we discuss how miRNAs influence cell apoptosis by affecting apoptosis initiators. In GC, many miRNAs directly modulate Bcl-2 expression. Downregulation of miR-15b, miR-16, miR-34, miR-181b [2], miR-204 [66], and miR-449a [67] promotes GC cell apoptosis by negatively regulating Bcl-2. Bcl-2 is also indirectly regulated by miRNAs. The downregulation of PTEN promotes the Akt signaling pathway, resulting in increased NF-κB. Furthermore, activated NF-κB upregulates the expression of Cox-2 [13], which reduces apoptosis by promoting Bcl-2 expression [68]. Interestingly, this chain reaction is observed in GC cells and is regulated by specific miRNAs. First, upregulated miR-21 [12] and miR-221/222 [2] can target *PTEN* directly. Meanwhile, NF-κB can induce miR-21 expression [15], thus forming a positive feedback loop between miR-21 and NF-κB. Second, the upregulation of miR-362 increases the activity and expression of NF-κB through inhibiting CYLD expression [16]. miR-218, which is reduced by Hp infection, inhibits NF-κB indirectly by targeting epidermal growth factor receptor-coamplified and overexpressed protein (ECOP) [69]. Finally, miR-101, which is decreased in GC, binds to the 3′-UTR of *Cox*-*2* mRNA, and inhibits Cox-2 expression directly [68]. In addition, miR-202-3p downregulation in GC can reduce Bcl-2 by inhibiting Gli1 [70]. Other Bcl-2 family members, such as Bax and Bid, are also regulated by miRNAs in GC. The transcription factor p53, which localizes in the mitochondria, can interact with Bak and Bax directly. However, this interaction blocks the interaction of Bak and Mcl-1. These effects change the mitochondrial permeability, resulting in the release of apoptosis factors and eventually leading to apoptosis [65]. In GC, many miRNAs can control cell apoptosis by regulating p53 and its downstream molecules. Upregulated miR-17-5p/20a decreases the expression of TP53INP1, thus weakening the role of p53 [30]. miR-449 reduction by Hp in GC induces apoptosis by inhibiting SIRT1, which represses the p53 pathway [26]. Upregulated miR-23a [71] directly targets IRF1, which mediates apoptosis by upregulating the expression of p53 upregulated modulator of apoptosis (PUMA). However, this is p53 independent [64]. miR-150 promotes GC cell proliferation by inhibiting EGR2, which enhances p53-mediated apoptosis and activates the proapoptotic proteins BNIP3L and Bak [2]. In addition, downregulated miR-101 promotes Bax expression directly [68], and miR-451 increases Bid- and Bax-mediated apoptosis by targeting macrophage migration inhibitory factor (MIF) [2]. Similarly, upregulated miR-25 and miR-130b inhibit the expression of Bim directly and indirectly, respectively, through decreasing RUNX3 [2]. Downregulated miR-29c [72], miR-512-5p [2], and miR-320 [73], which is inhibited by Hp, can suppress the expression of Mcl-1. In addition to this intrinsic pathway, miR-106a upregulated in GC represses the extrinsic pathway by targeting the 3′-UTR of Fas directly [74].

### ncRNAs affects apoptosis effectors

Next, we discuss the influence of miRNAs on apoptosis effectors (Fig. 3). By DNA methylation, miR-375 silencing in GC can induce apoptosis in two ways. First, miR-375 can inhibit the PDK1/Akt/XIAP pathway. Downstream of Akt, XIAP inhibits apoptosis by suppressing caspase activity. Second, miR-375 targets the 3′ UTR of 14-3-3*ζ*, which inhibits proapoptotic proteins such as Bad and JAK. Both siPDK1 and si14-3-3ζ transfectants increased caspase-3/7 activity [75]. In addition to miR-375, many other miRNAs can also affect apoptosis through the Akt pathway. miR-125-5p and miR-146a are downregulated in GC, and both regulate Akt by directly targeting the *EGFR* mRNA [2]. As mentioned above, PTEN is a negative regulator of PI3K-Akt signaling. The miR-221/222 cluster [2] and miR-21 [12] block apoptosis of GC cells by targeting *PTEN* mRNA directly. miR-338 promotes apoptosis and regulates the phosphorylation of Akt by inhibiting neuropilin-1 (NRP1) [19]. Beyond these classic indirect ways, caspase-3, a direct effector of apoptosis, is also activated by miRNAs. The downregulation of miR-409-3p results in the overexpression of PHF10 in GC. PHF10 inhibits cell apoptosis by binding to the promoter region of *caspase*-*3* directly [76]. The miR-17-92 cluster encodes miR-18a, which can promote STAT3 and its downstream effectors including Myc, survivin, and Bcl-xl through inhibiting PIAS3, which negatively regulates STAT3. Bcl-xl is anti-apoptotic, and survivin inhibits the release of Cyt c and the level of caspase-3 [77]. Therefore, miR-18a may block the apoptotic process [56].

Interestingly, unlike miRNAs, HULC upregulation in GC tissues and cell lines inhibits cell apoptosis by activating autophagy, which has a dual role in cancer development [78].

## ncRNAs associated with invasion and metastasis in GC

Tumor invasion and metastasis are complex and multistep processes. Here, we discuss three aspects: (1) the alteration of cell phenotypes including the decrease of epithelial cell marker genes such as E-cadherin, the increase of mesenchymal cell marker genes such as *N*-*cadherin* and *integrin* [79], and changes in tumor cell motility and shape [80]—these conversions are also called epithelial-mesenchymal transition (EMT); (2) the remodeling of the extracellular matrix (ECM), which requires matrix metalloproteinases (MMPs) and other proteolytic enzymes [2], and (3) the proliferation of neovascularization, which contributes to an invasive and metastatic tumor microenvironment [79].

EMT is induced by several signaling pathways. Transforming growth factor beta (TGFβ) is the most potent and most well-described inducer. Others like E-cadherin transcriptional repressors also act as EMT inducers, including the Snail, zebra (ZEB), and Twist families [81]. TGFβ1 initiates EMT by activating either the Smad 2/3/4 trimer or non-Smad pathways, including PI3K/Akt, RAS small GTPases, and Wnt/β-catenin. Many of these pathways work synergistically in EMT. For instance, TGFβ-mediated Smad3/4 promote Snail and ZEB1/2 expression [80], and the activation of GF/TGFβ-Ras-Akt signaling increases the expression of Snail, Twist, Slug, and Smad [17]. The stimulation of myosin light chain phosphorylation and actin reorganization can potentiate TGFβ-induced EMT. β-catenin signaling, which is enhanced by TGFβ [80] and inhibited by Akt [17], can mediate the binding of E-cadherin to the actin cytoskeleton and regulate the expression of Snail and Slug [80]. It is noteworthy that NF-κB has been identified as a key regulator of EMT as it can induce the expression of Snail and ZEB1/2. In addition, EMT is regulated by cytokines and integrin signaling [80].

There is no doubt that the mechanisms of tumor invasion and metastasis are complex. Interestingly, both miRNAs [82] and lncRNAs [5] participate in cancer metastasis in GC [2, 6].

### ncRNAs associated with EMT in GC

#### Effect of miRNAs and lncRNAs on signaling upstream of EMT

**Fig 4:**
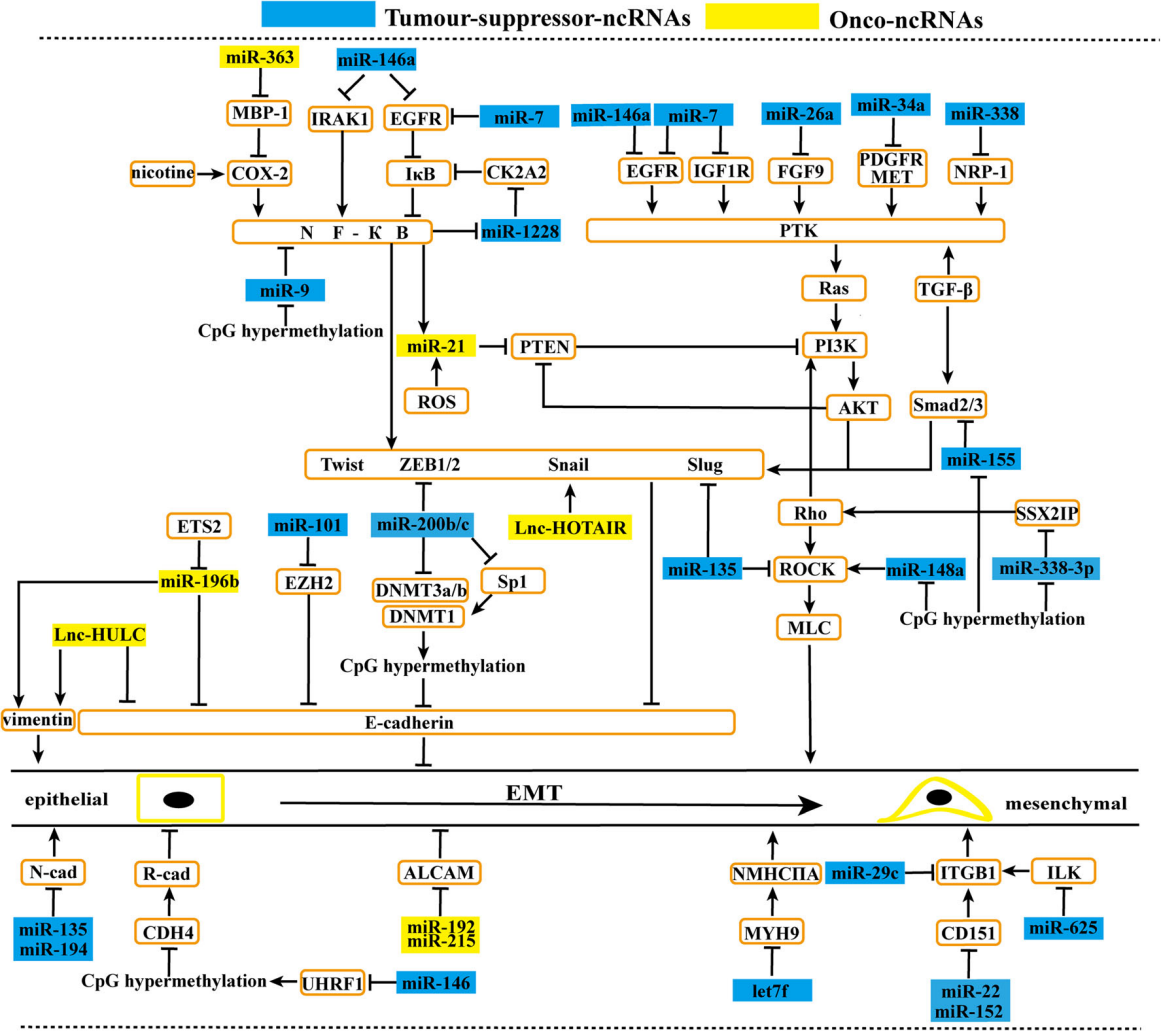
ncRNAs associated with EMT in GC. MiRNAs and lncRNAs affect gastric cancer cell metastasis. By regulating EMT, onco-ncRNAs promote gastric cancer cell metastasis. Conversely, tumor suppressor ncRNAs suppress gastric cancer cell metastasis

The GF-Ras-PI3K-Akt pathway plays a key role in EMT [17]. In GC, many miRNAs regulate EMT by affecting GFs or their receptors and Akt signaling (Fig. 4). miR-146a [83] and miR-7 [84] suppress EGFR. Meanwhile, miR-7 is inversely correlated with insulin-like growth factor-1 receptor (IGF1R) [85]. miR-26a attenuates FGF9 [86], miR-34a inhibits PDGFR-a/b [87], and miR-338 decreases NRP1, a receptor for the vascular endothelial growth factor (VEGF) [19]. All five miRNAs are downregulated in GC and can inhibit cell migration. TGFβ not only activates the Ras-PI3K-Akt pathway [17] but also promotes EMT by regulating Smad2/3 [80]. Surprisingly, S-S Lo et al. reported that miR-370 increased the migration of GC cells by disrupting TGFβ signaling [88]. However, TGFβ is not the only way to regulate Smads. miR-155, which is downregulated by DNA methylation, may inhibit Smad2 expression by targeting its 3′UTR [89]. In addition, ROS promotes the expression of miR-21 in GC [14]. In turn, upregulated miR-21 inhibits PTEN [12], reducing its inhibitory effect on Akt [17]. Furthermore, Cox-2 stimulated by nicotine can promote miR-21 expression via activating NF-κB, which targets miR-21 directly [15]. Thus, a complex network forms among NF-κB, Akt, and EMT by regulating miRNAs.

NF-κB not only targets miRNAs but is also regulated by many miRNAs in GC (Fig. 4). As an oncomir, miR-363 [60] enhances EMT through inhibiting MBP-1, which blocks Cox-2 expression. Consistently, the upregulation of Cox-2 activates NF-κB/Snail signaling but decreases E-cadherin expression [90]. The reduction of miR-146a is associated with the upregulation of EGFR and IRAK1. IRAK1 is upstream of NF-κB, and EGFR activates NF-κB by attenuating IκB [83]. miR-1228* is downregulated and forms a negative feedback loop with NF-κB through targeting CK2A2 expression, which degrades IκB [91]. DNA hypermethylation can result in low miR-9 expression in GC. Dysregulated miR-9 affects cell metastasis in two ways. First, it inhibits NF-κB1. Second, it decreases the expression of MMP2, MMP9, Twist, and N-cadherin [48].

#### Direct effects of miRNAs and lncRNAs on EMT-TFs or the phenotype of GC cells

In addition to affecting the signaling upstream of EMT, a host of miRNAs and lncRNAs can also directly regulate the EMT-TFs or the phenotype of GC cells (Fig. 4). miR-101, which is downregulated in GC, increases the expression of E-cadherin through inhibiting EZH2 [92]. In GC, lack of ETS2 causes the upregulation of miR-196b, which increases the expression of vimentin, MMP2, and MMP9, but decreases E-cadherin [93]. The miR-200 family, including miR-200a/b/c, is downregulated in GC. During EMT, they not only negatively regulate *ZEB1*/*2* [94] but also inhibit DNA methyltransferases (DNMTs), leading to the hypomethylation of promoter DNA and upregulation of E-cadherin [39]. Furthermore, miR-135a downregulation in GC can increase E-cadherin expression by suppressing Slug expression and inhibiting N-cadherin expression [95]. Song et al. reported that with the transfection of miR-194 mimics, both the expression of N-cadherin and the metastasis of GC cells were suppressed [96]. miR-146a/b downregulation in GC can regulate metastasis by reducing the expression of epigenetic regulator ubiquitin-like containing PHD ring finger 1 (UHRF1), which maintains DNA methylation by recruiting DNMT1. DNA hypermethylation can silence *CDH4* and *RUNX3. CDH4* encodes R-cadherin, and Runx3 inhibits MMP9 via upregulating TIMP-1 [97]. Both miR-192 and miR-215 are upregulated in GC and can significantly decrease the expression of ALCAM, a cell adhesion molecule expressed by epithelial cells [98]. Like miRNAs, lncRNAs play important roles in EMT. HULC is overexpressed in GC, and knockdown of *HULC* can downregulate vimentin and upregulate E-cadherin expression [78]. In addition, HOTAIR, which is also upregulated in GC, promotes EMT by stimulating the expression of Snail, MMP1, and MMP3 [99].

#### Effect of miRNAs and lncRNAs on cancer cell motility

During EMT, cancer cell motility also changes greatly and is regulated by many ncRNAs (Fig. 4). Accumulating evidence has reported that Rho [100] promotes cell invasion by regulating the PI3K/Akt and ROCK signaling pathways, which promote actomyosin contractility via mediating the phosphorylation of MLC, significantly contributing to cell motility [101, 102]. Epigenetically silenced miR-338-3p can inhibit the expression of Rac1, a Rho family member, by reducing SSX2IP expression [103]. Both downregulated miR-135 [95] and miR-148a [102], which is silenced by DNA hypermethylation [104], target ROCK1 directly. PINCH, integrin-linked kinase (ILK) and parvin work as a PINCH–ILK–parvin (PIP) complex. PIP complexes provide crucial physical linkages between integrins and the actin cytoskeleton and transduce signaling from the ECM to intracellular effectors. ILK contains many distinct integrin binding sites. It is therefore assumed that an integrin β-IPP-actin pathway is involved in abnormal cell-ECM adhesion and cell motility [105]. In GC, downregulated miR-625 [106] and miR-29c [107] can target ILK and integrin β, respectively. However, miR-22 [108] and miR-152 [109] suppress GC cell motility partially by inhibiting CD151, which contributes to integrin-mediated metastasis. Let-7f inhibits and binds to the 3′-UTR of *MYH9*, which codes for myosin IIA directly, leading to an attenuation of cell motility [110].

### Degradation of ECM by ncRNAs

**Fig 5:**
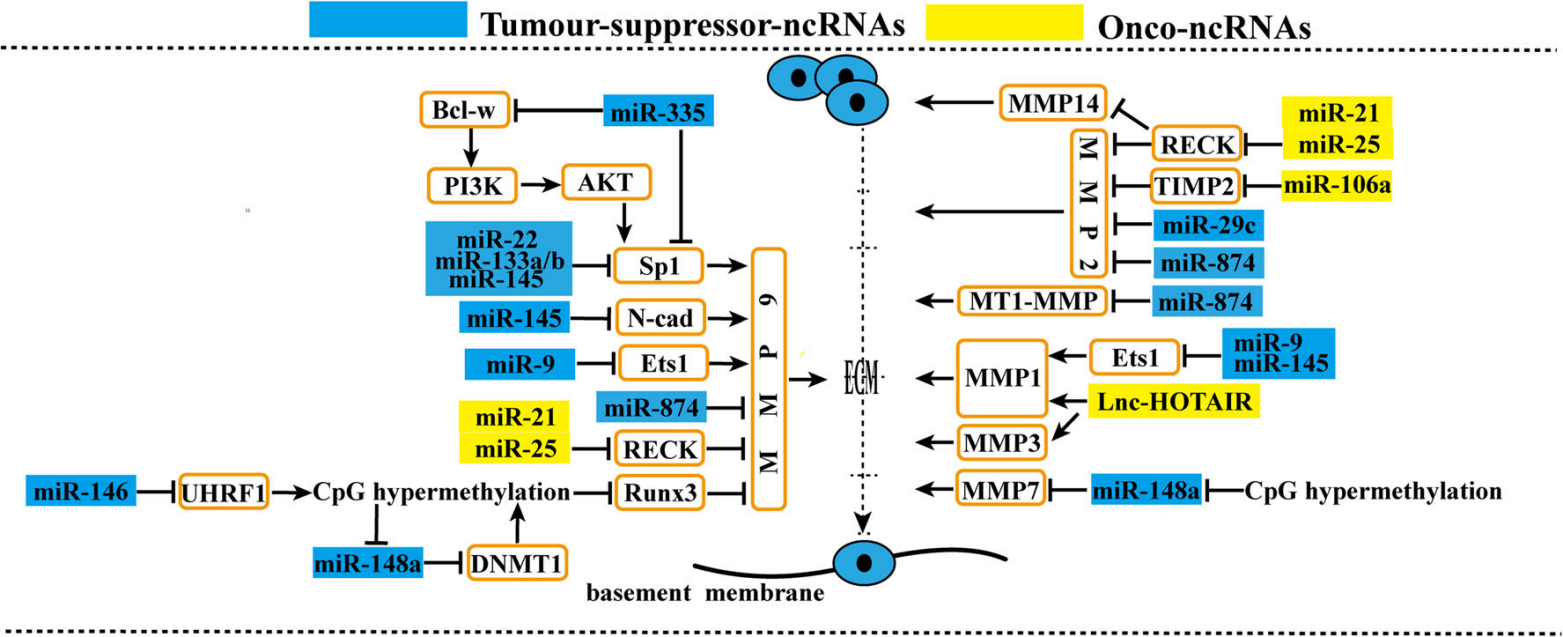
Degradation of ECM by ncRNAs in GC. MiRNAs and lncRNAs affect gastric cancer cell metastasis. By regulating remodeling of ECM, onco-ncRNAs promote gastric cancer cell metastasis. Conversely, tumor suppressor ncRNAs suppress gastric cancer cell metastasis

The degradation of the ECM is a crucial step in the progression of tumor metastasis. MMPs hydrolyze type IV collagen and promote cell invasion [111]. Similarly, MMPs are also inhibited or activated by many signaling molecules and ncRNAs (Fig. 5). Bcl-w promotes GC metastasis by activating the PI3K-Akt-Sp1 pathway. miR-335, which is downregulated in GC, prevents cell invasion and metastasis by targeting specificity protein 1 (Sp1) directly, and indirectly through regulating Bcl-w [47]. miR-22, miR-133a, miR-133b, and miR-145 are also downregulated in GC and can inhibit metastasis via negatively regulating Sp1 [45, 112]. Meanwhile, Qiu et al. reported that in GC, knockdown of *Sp1* reduced the expression of MMP-9 [45]. MMP-9 may therefore be the common effector of the five miRNAs. N-cadherin also acts as an upstream promoter of *MMP9* and is directly inhibited by miR-145, which is downregulated in GC [113]. In addition, both *MMP1* and *MMP9* are downstream of v-ets erythroblastosis virus E26 oncogene homolog 1 (Ets1), and Ets1 is suppressed by miR-145 [114] and miR-9 [48, 49] downregulation in GC. As a member of the tissue inhibitors of metalloproteinases (TIMPs) family, TIMP2 can combine with MMP2 directly and work as downstream of miR-106a, which is upregulated in GC [115]. However, miR-21 [2] and miR-25 [116], which are also elevated in GC, can suppress reversion-inducing cysteine-rich protein with kazal motifs (RECK), a new MMP inhibitor, which simultaneously inhibits MMP-2, MMP-9, and MMP14. miR-874 can suppress MT1-MMP, MMP-2, and MMP-9, and these inhibitory effects may be dependent upon AQP3 [117]. In addition to these indirect effects, many miRNAs and lncRNAs act as direct regulators of MMPs in GC. For example, miR-29 targets *MMP2* [41], and miR-148a targets *MMP7* [118]. Furthermore, upregulated HOTAIR can induce the expression of MMP1 and MMP3 [99].

### Angiogenesis by ncRNAs

**Fig 6:**
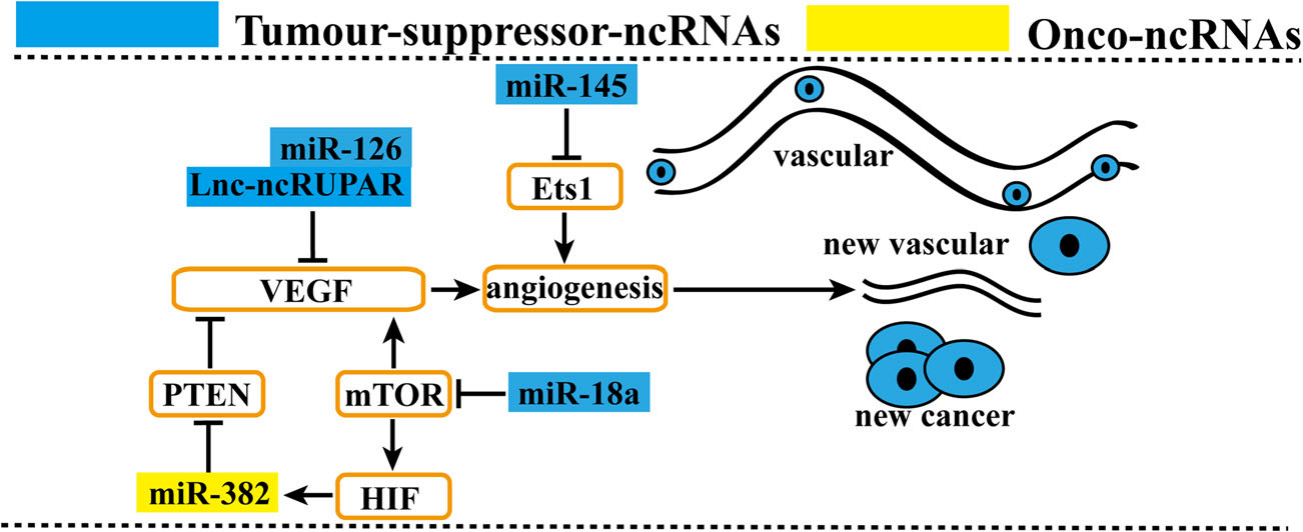
Angiogenesis by ncRNAs in GC. MiRNAs and lncRNAs affect gastric cancer cell metastasis. By regulating angiogenesis, onco-ncRNAs promote gastric cancer cell metastasis. Conversely, tumor suppressor ncRNAs suppress gastric cancer cell metastasis

Tumor-associated endothelial cells, composing the arteries, veins, and capillaries, are prominent tumor stromal constituents and play key roles in cell metastasis [79]. Although the impact is not so great as on EMT, some ncRNAs affect the development of tumor-associated angiogenesis (Fig. 6). miR-126, which was downregulated, facilitated GC angiogenesis by regulating VEGF-A [119]. miR-382 was induced by hypoxia, promoted angiogenesis, and acted as an angiogenic oncogene by repressing PTEN, which inhibited miR-382-induced angiogenesis and VEGF secretion [120]. miR-18a overexpression significantly reduced tumor angiogenesis and substantially reduced the inactivation of the mTOR pathway. Accompanying mTOR inactivation, the angiogenic factors hypoxia-inducible factor 1 alpha and vascular endothelial growth factor were significantly downregulated [121]. miR-145 was downregulated and suppressed Ets1 expression via the binding site in the 3′-UTR, thus inhibiting the invasion, metastasis, and angiogenesis of gastric cancer cells [114]. RuPAR is reduced and inversely associated with the expression of VEGF protein in GC tissues. Moreover, both are significantly correlated with invasion depth, lymph node metastasis, and distant metastasis [122]. Therefore, we speculated that ruPAR promotes GC metastasis by stimulating angiogenesis.

## Conclusion

The molecular mechanisms of miRNAs and lncRNAs in gastric carcinogenesis are much more complex than we discussed in this paper. Functional analyses have shown that these miRNAs and lncRNAs interact with mRNAs from oncogenes and tumor suppressor genes. The altered expression of ncRNAs in GC promotes cell cycle progression via direct and indirect regulation of Akt pathways, CDKIs or Myc, reduces apoptotic signaling through the regulation of apoptosis initiators and apoptosis effectors, and promotes cell migration and invasion by regulating EMT pathways, degradation of ECM by ncRNAs or angiogenesis. They work in a network rather than as individuals. One ncRNA can affect several biological behaviors, including the cell cycle, apoptosis, and metastasis, through several signaling pathways. Meanwhile, one signaling pathway can be affected by several ncRNAs and can regulate several biological behaviors. Therefore, blocking only one of these functional sites or ncRNAs may not stop the progress of gastric tumorigenesis. These novel mechanisms of miRNA and lncRNAs not only help us elucidate the pathogenesis of GC, but also offer us opportunities for ncRNA-targeting strategies. However, the great complexity of these mechanisms also brings a huge challenge to the study of ncRNAs in GC.
